# Thermoregulatory postures limit antipredator responses in peafowl

**DOI:** 10.1242/bio.031005

**Published:** 2018-01-15

**Authors:** Jessica L. Yorzinski, Jennifer Lam, Rachel Schultz, Melissa Davis

**Affiliations:** 1 Department of Wildlife and Fisheries Sciences, Texas A&M University, College Station, TX 77843-2258, USA; 2 Department of Biological Sciences, Purdue University, 915 West State Street, West Lafayette, IN 47907, USA; 3 Department of Animal Sciences, Purdue University, 915 West State Street, West Lafayette, IN 47907, USA

**Keywords:** Antipredator behavior, Peafowl, Thermoregulation

## Abstract

Many animals inhabit environments where they experience temperature fluctuations. One way in which animals can adjust to these temperature changes is through behavioral thermoregulation. However, we know little about the thermal benefits of postural changes and the costs they may incur. In this study, we examined the thermoregulatory role of two postures, the head-tuck and leg-tuck posture, in peafowl (*Pavo cristatus*) and evaluated whether the head-tuck posture imposes a predation cost. The heads and legs of peafowl are significantly warmer when the birds exhibit these postures, demonstrating that these postures serve an important thermoregulatory role. In addition, the birds are slower to respond to an approaching threat when they display the head-tuck posture, suggesting that a thermoregulatory posture can limit antipredator behavior.

## Abstract (precis)

**Summary:** The head-tuck and leg-tuck postures of peafowl help conserve heat but can impair antipredator responses.

## INTRODUCTION

Many animals live in environments with temperature fluctuations throughout the year. One way that animals can attempt to counter the negative effects of fluctuating temperatures is through thermoregulatory mechanisms (Kearney et al., 2009). These thermoregulatory mechanisms can help keep animals within their thermoneutral zones (Taylor, 1986). Animals across many different taxa use morphological, physiological, and behavioral adaptations to adjust to variations in environmental temperature (Taylor, 1986; Wright, 1994; Fortin et al., 2000; Seebacher and Franklin, 2005; Briscoe et al., 2014; Ancel et al., 2015).

When animals use behavioral strategies to thermoregulate, they can rely on conspecifics. Emperor penguins, for example, conserve heat by huddling with each other (Ancel et al., 2015). Alternatively, animals using behavioral strategies for thermoregulation can locate themselves in specific microhabitats. For example, koalas rest on cool trees to dissipate heat (Briscoe et al., 2014) and goslings orient toward the sun (and away from the wind) to stay warm (Fortin et al., 2000). In addition, animals can adopt specific postures to thermoregulate, but we know little about the thermal benefits of them doing so. Birds tuck their heads under their wings (Deighton and Hutchinson, 1940; Veghte and Herreid, 1965), and tuck their legs under their bodies (Carr and Lima, 2012), in cold temperatures; and koalas expose more surface area of their bodies when it is warmer (Briscoe et al., 2014).

These thermoregulatory mechanisms help animals cope with fluctuating environmental temperatures but may also impose costs (Huey and Slatkin, 1976). For example, mourning doves have a reduced ability to fly when they are hypothermic at night (Carr and Lima, 2013). In willow warblers, they likely expend more energy singing in cold versus warmer microhabitats (Ward and Slater, 2005). Dark-eyed juncos' ability to escape from a threat is hindered when they display more heat-conserving postures (Carr and Lima, 2012). While we are beginning to understand the costs associated with thermoregulatory mechanisms, additional studies are still needed to further understand trade-offs associated with thermoregulation.

**Fig. 1.**
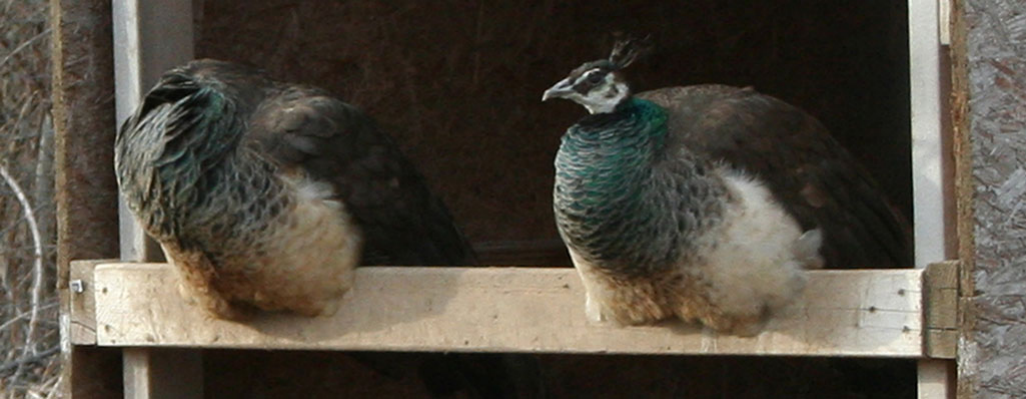
**A peahen in the head-tuck (left) and upright (right) posture.** Both birds are in the leg-tuck posture.

We therefore studied the thermoregulatory role of postures and investigated whether they impose a predation cost. In particular, we examined the head-tuck and leg-tuck posture (Fig. 1) of Indian peafowl (*Pavo cristatus*). Peafowl are native to the Indian subcontinent and can therefore experience hot summers and cold winters depending on an individual's exact geographic location (Dodsworth, 1912; Ali and Ripley, 1969). When birds are exhibiting the head-tuck posture, they conceal their head under their wing, and when they are exhibiting the leg-tuck posture they conceal their feet and legs under their bodies; they adopt these postures during the day and night. First, we assessed the thermal benefits of these postures by quantifying the temperatures of the birds exhibiting these postures, using both temperature sensors and infrared thermography. In other birds (including galliformes), skin surface temperature and body temperature are highly correlated (Giloh et al., 2012; Ikkatai and Watanabe, 2015). Second, we tested whether one of the postures (head-tuck posture) limited the ability of the birds to respond to an approaching threat.

## RESULTS

**Fig. 2.**
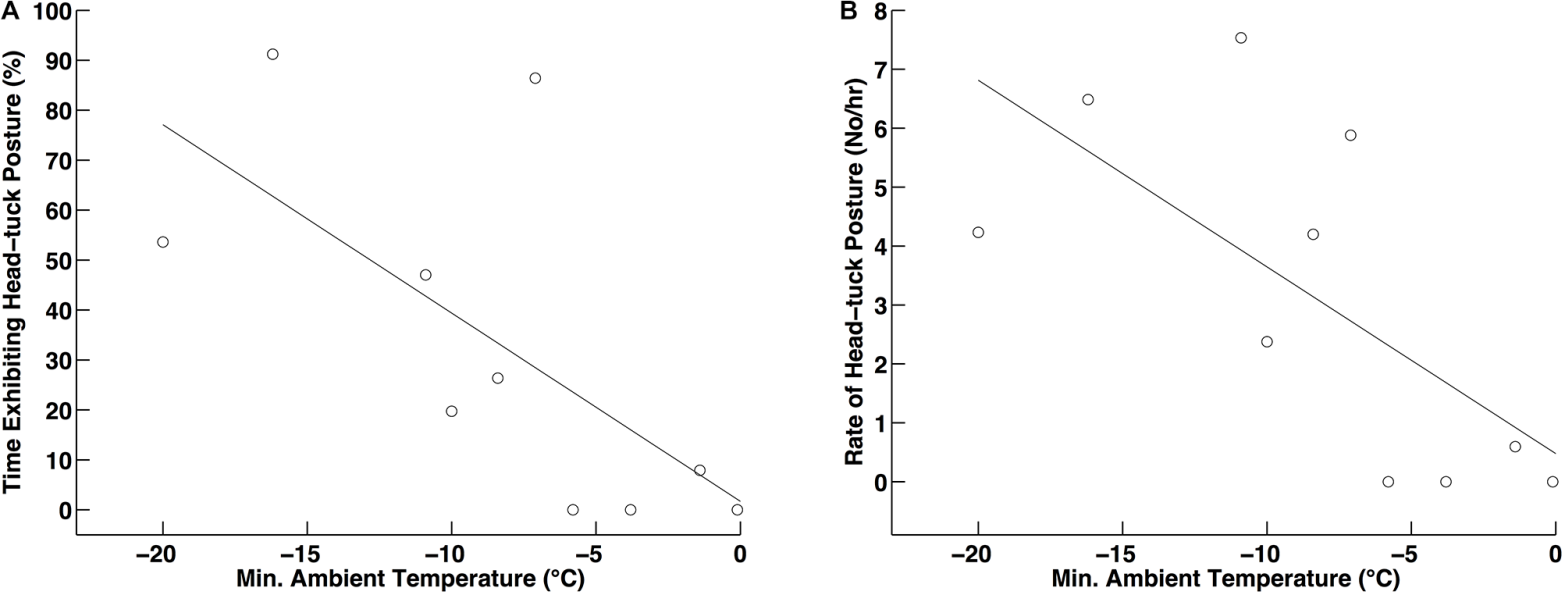
**The effect of ambient temperature on the head-tuck posture.** (A) The amount of time and (B) rate that birds exhibited the head-tuck posture with respect to ambient temperature.

Environmental variables impacted when birds exhibited the head-tuck posture but not the leg-tuck posture. Birds spent more time in the head-tuck posture (*F*_1,7_=6.00, *P*=0.044) and exhibited a higher rate of head-tuck postures (*F*_1,7_=6.33, *P*=0.040; *n*=10) on colder nights (Fig. 2). Wind speed did not impact the amount of time that birds spent in the head-tuck posture (*F*_1,7_=0.20, *P*=0.67) nor the rate of head-tuck postures (*F*_1,7_=0.47, *P*=0.52). In contrast, the ambient temperature and wind speed did not impact the amount of time (temperature: *F*_1,10_=0.00, *P*=0.97; wind: *F*_1,10_=0.05, *P*=0.82) or the rate that birds spent in the leg-tuck posture (temperature: *F*_1,10_=0.00, *P*=0.96; wind: *F*_1,10_=0.56, *P*=0.47; *n*=13).

**Fig. 3.**
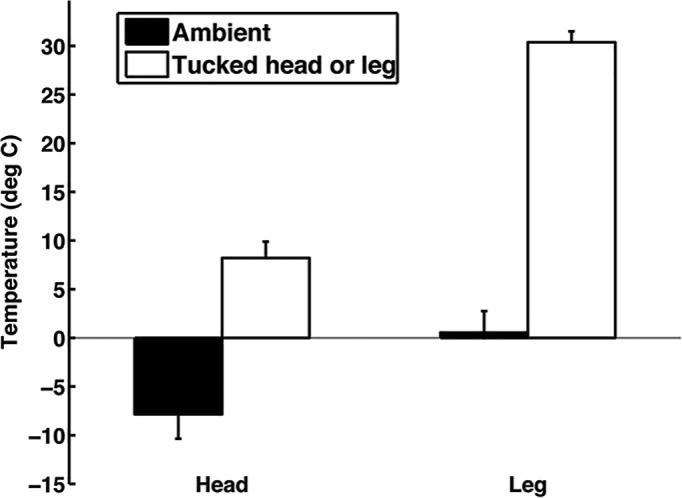
**The temperature of the heads and legs in the head-tuck and leg-tuck postures, respectively, relative to ambient temperature.** Error bars represent mean±s.e.m.

Based on the temperature sensor analysis, the head-tuck and leg-tuck postures help conserve heat. The temperature of the birds' heads was higher than ambient temperature when the birds exhibited the head-tuck posture (t=12.0, *n*=7, *P*<0.0001; Fig. 3). The temperature difference between the birds' heads in the head-tuck posture and ambient temperature was greater when the duration of the head-tuck was longer (*F*_1,435_=31.23, *P*<0.0001) and the ambient temperature was colder (*F*_1,435_=36.26, *P*<0.0001; *n*=7). Similarly, the temperature of the birds' legs was higher than ambient temperature when the birds exhibited the leg-tuck posture (t=17.0, *n*=13, *P*<0.0001). The temperature difference between the birds' legs in the leg-tuck posture and ambient temperature was greater when the duration of the leg-tuck was longer (*F*_1,35_=7.1, *P*=0.012) and the ambient temperature was colder (*F*_1,35_=22.65, *P*<0.0001), but the sex of the bird did not impact this temperature difference (*F*_1,35_=0.00, *P*=0.99; *n*=13). For a given bout, birds remained in the leg-tuck posture (11,024±1823 s) longer than the head-tuck posture (381±26 s). While birds usually exhibited the head-tuck posture when they were in the leg-tuck posture (97.7% of head-tuck bouts occurred when the birds were sitting; based on the head-tuck trials), birds sometimes exhibited the head-tuck posture while standing.

**Fig. 4.**
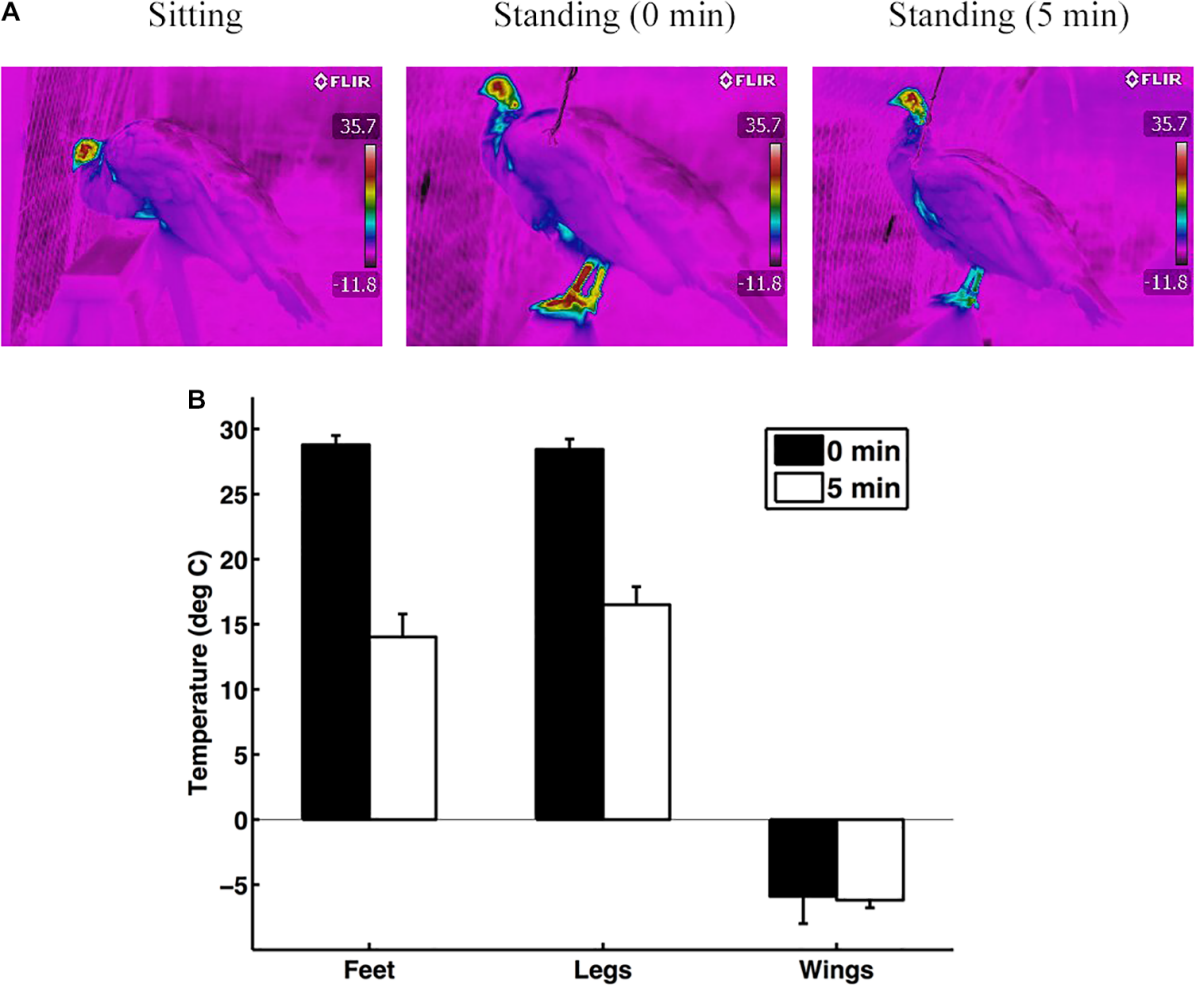
**The effect of the leg-tuck posture on heat conservation.** (A) Thermal images of a peahen sitting in the leg-tuck posture, immediately after she stands up from the leg-tuck posture (0 min), and 5 min after she stands up from the leg-tuck posture. (B) The temperature of the feet, legs, and wings immediately after birds stood up after being in the leg-tuck posture (0 min) and after the birds remained standing (5 min). Error bars represent mean±s.e.m.

Based on the thermal image analysis, the leg-tuck posture helps conserve heat. The temperature of the birds' feet and legs was higher immediately after the birds stood up from the leg-tuck posture compared to after they remained standing for five minutes (feet: *F*_1,6_=70.10, *P*=0.0002; legs: *F*_1,6_=72.57, *P*=0.0001; *n*=7; Fig. 4). The sex of the bird (feet: *F*_1,6_=2.74, *P*=0.15; legs: *F*_1,6_=0.11, *P*=0.75), wind speed (feet: *F*_1,6_=0.96, *P*=0.37; legs: *F*_1,6_=0.04, *P*=0.85), ambient temperature (feet: *F*_1,6_=0.02, *P*=0.89; legs: *F*_1,6_=0.01, *P*=0.93), and interaction between wind speed and ambient temperature (feet: *F*_1,6_=0.29, *P*=0.61; legs: *F*_1,6_=0.00, *P*=0.98) did not impact the surface temperature of the birds' feet or legs. As expected, the surface temperature of the birds' wing was unrelated to whether the bird was in the leg-tuck posture or not (Fig. 2; *F*_1,6_=0.14, *P*=0.72) or the sex of the bird (*F*_1,6_=0.55, *P*=0.49). However, the surface temperature of the birds' wings was higher when the wind speed was lower (*F*_1,6_=14.08, *P*=0.0095) and the ambient temperature was higher (*F*_1,6_=21.29, *P*=0.0036). The interaction between wind speed and ambient temperature also impacted wing surface temperature (*F*_1,6_=42.80, *P*=0.0006). Comparing the thermal image and temperature sensor methods, the temperature of the birds' legs immediately after the birds stood up from the leg-tuck posture (recorded with the infrared thermography) was similar to the temperature of the birds' legs while they were in the leg-tuck posture (recorded with the temperature sensor; *F*_1,15_=0.81, *P*=0.42).

**Fig. 5.**
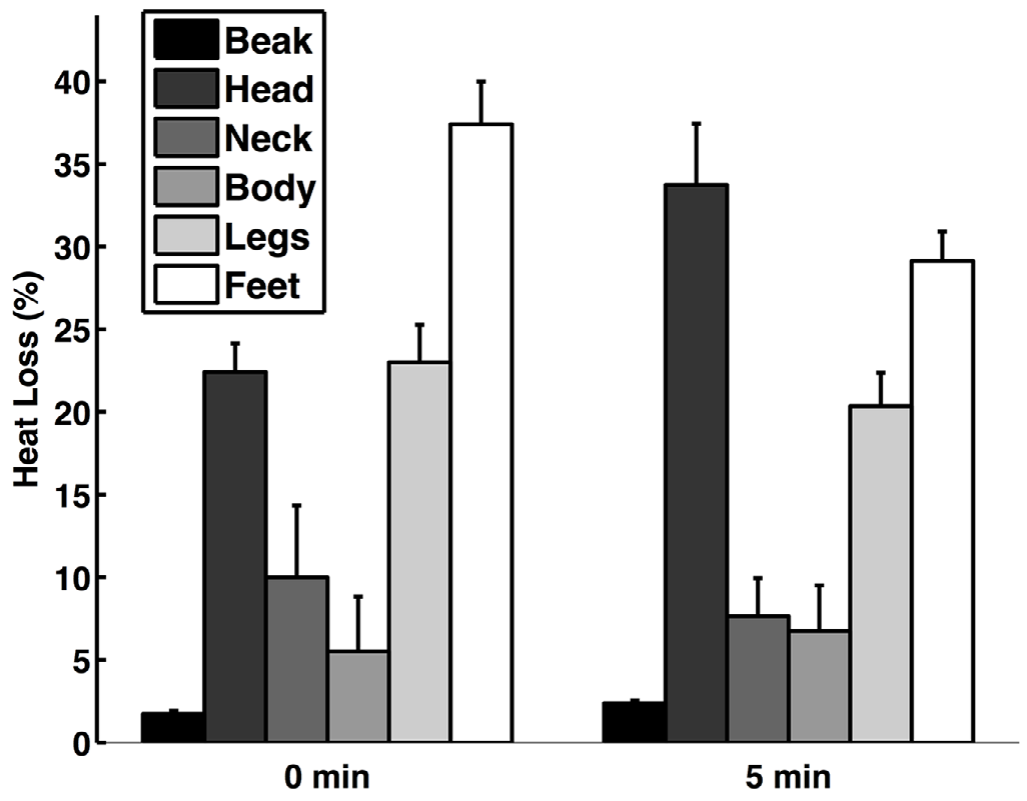
**Heat loss (%) of morphological regions depending on whether the birds initially stood up from the leg-tuck posture (0 min) or after they remained standing for 5 min.** Error bars represent mean±s.e.m.

Analysis of the thermal images revealed that heat loss was not uniform but varied across morphological regions. Immediately after birds stood up from the leg-tuck posture, they were losing the highest percentage of heat from their feet (38%); after they remained standing for 5 min, they were losing the highest percentage of heat from their heads (33%; Fig. 5). The amount of heat loss varied based on whether the birds immediately stood up or were standing for 5 min (*F*_1,66_=5.91, *P*=0.018) and on morphological region (*F*_5,66_=27.22, *P*<0.0001). The amount of heat lost through their feet decreased after the birds were standing for 5 min (t=3.56, d.f.=66, *P*=0.0007) and the amount of heat lost through their legs tended to decrease after the birds were standing for 5 min (t=1.63, d.f.=66, *P*=0.11) compared to when they initially stood up from the leg-tuck posture. Heat loss did not differ among other morphological regions (beak, head, body, and neck) between when the birds initially stood up and 5 min afterwards (*P*>0.42). As expected, heat loss was higher when wind speed was higher (*F*_1,66_=17.68, *P*<0.0001).

**Fig. 6.**
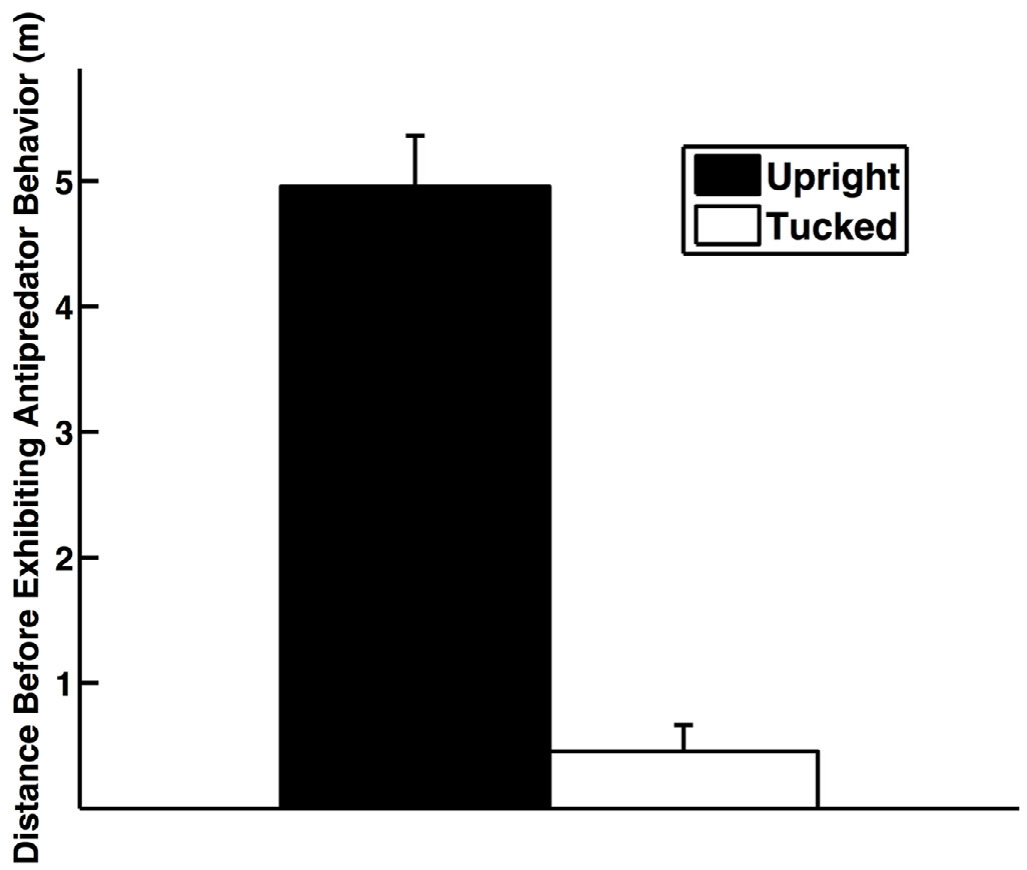
**Distance that a potential threat approached birds in the head-tuck posture versus birds that held their heads upright.** Error bars represent mean±s.e.m.

One of the postures (head-tuck posture) impacts antipredator behavior. The researcher approached birds in the head-tuck posture closer than birds that held their heads upright (Fig. 6; Mann–Whitney test statistic=126, *n*=18, *P*=0.0004). In fact, the researcher was able to approach 56% of the birds in the head-tuck posture without the birds detecting the researcher standing directly beside them (they remained with their heads tucked under their wings); in contrast, the researcher was only able to approach to within 2.6 m of any bird that held its head upright. Even though the birds were in captivity, they still clearly viewed humans as threats given that they did not allow the experimenter to approach very closely when their heads were upright.

## DISCUSSION

Peafowl adopt postures to regulate their temperature in cold environments. They exhibit a head-tuck posture in which they put their heads underneath their wings and a leg-tuck posture in which they conceal their legs and feet underneath their bodies. The temperature of their heads and legs is significantly warmer when the birds exhibit these postures versus when they leave these extremities exposed. Furthermore, when the birds use the head-tuck posture, they are slower to respond to an approaching threat compared to when their heads are upright.

Heat loss is not uniformly distributed across morphological regions in birds. The head is often the region where most heat is lost (Veghte and Herreid, 1965; Hill et al., 1980; Phillips and Sanborn, 1994; McCafferty et al., 1998; Greenberg et al., 2012). For example, in sharp-tailed grouse and barn owls, the heads lose 2-3 times more heat than other body areas even though the surface area of the heads is relatively small (Evans and Moen, 1975; McCafferty et al., 1998). The legs and feet are also poorly insulated (Johansen and Bech, 1983) but countercurrent blood flow can allow birds to minimize heat loss from these extremities (Kilgore and Schmidt-Nielsen, 1975; Greenberg et al., 2012). The head-tuck and leg-tuck postures that peafowl adopt target these poorly insulated body areas. The head-tuck posture in peafowl conserves their superficial head temperature around 8°C despite the ambient temperature dropping below zero (we only tested females in this head-tuck experiment and future experiments would be necessary to assess whether any difference between the sexes exist). Given that the lower critical temperature of the thermoneutral zone in other galliformes is near zero (Rasmussen and Brander, 1973; Rintamäki et al., 1983), this head-tuck posture would elevate the temperature of the peafowls' head above this critical temperature. Similarly, the leg-tuck posture also maintains the legs of the birds at high temperatures. The legs are around 30°C when the ambient temperature is near zero. We are unaware of other studies that have experimentally quantified the thermal benefits of different postures, but others have suggested that these postures help conserve heat (Deighton and Hutchinson, 1940; Veghte and Herreid, 1965; Carr and Lima, 2012). Because surface area influences the amount of heat loss, postures that alter the amount of surface area that animals expose theoretically have thermal benefits (Briscoe et al., 2014).

Both of the methods (temperature sensor and infrared thermography) that we used to record the leg temperature of birds in the leg-tuck posture produced similar results (the legs are warmed to around 30°C in the leg-tuck posture). The temperature sensor method enabled us to continuously monitor the birds' temperature without disturbing them by our presence (aside from when we attached and removed the sensors). It also allowed us to record their temperature in areas that were not visible to us (under the feathers). The infrared thermography method was more limiting in that we needed to be close to the animals in order to take the thermal images and our presence therefore disturbed the birds. However, when it is not possible to attach sensors onto animals, this infrared thermography method would be useful in understanding the thermoregulatory role of body postures given that the results from the two methods were similar.

Despite the thermal benefits of the head-tuck posture, peafowl were more susceptible to threats when they exhibited this posture. By tucking their heads under their wings, they lose visual contact with the environment and may also have reduced auditory abilities because their ears are covered by feathers. It is likely that this loss of visual information is especially significant during daylight but may also be important at night when threats are at close range (Martin, 1993). In over half of the trials, a threat was able to approach immediately beside birds that exhibited the head-tuck posture during the day. If the threat were an actual predator, a bird in the head-tuck posture (whether asleep or awake) would have had little opportunity to escape once the predator initiated an attack (Ydenberg and Dill, 1986). Because we were not monitoring sleep behavior (e.g. by using an electroencephalogram), it is possible that birds in the head-tuck posture were sleeping and therefore generally less alert than birds that held their heads upright (with their eyes open). However, it is unlikely that birds were in REM sleep (Tobler and Borbély, 1988) during this experiment as the experimenter approached the birds within 5 min of them initially exhibiting the head-tuck posture and these trials were performed during daylight. Future experiments that record sleep states when birds exhibit these different postures would be informative. Several other studies have found a similar trade-off between thermoregulatory and antipredator behaviors. Dark-eyed juncos adopting postures during foraging that better conserve heat (tucking their feet and legs under their feathers versus having their feet and legs exposed) are slower to exhibit a flight response. This slower response likely results from the birds needing to reposition themselves (e.g. putting both feet on the ground) before taking flight (Carr and Lima, 2012). Mourning doves become hypothermic at night to conserve heat but this impairs their ability to fly (Carr and Lima, 2013); and short-toed treecreepers gain thermal benefits when foraging on sunlit tree trunks compared to shaded tree trunks, but are more conspicuous to predators on the sunlit trunks (Carrascal et al., 2001).

Because of this potential predation cost, it is likely beneficial for animals to selectively exhibit thermoregulatory behaviors. In fact, the peafowl were selective in when they used the head-tuck posture. They were less likely to exhibit the head-tuck posture on warm nights. Similarly, turkeys also perform the head-tuck posture and are more likely to adopt this posture when their body temperature is low (Buchholz, 1996). Furthermore, dark-eyed juncos (Carr and Lima, 2012) and short-toed treecreepers (Carrascal et al., 2001) are less likely to display heat-conserving thermoregulatory behaviors when the ambient temperature is high. In contrast, peafowl used the leg-tuck posture throughout the majority of nights regardless of temperature. While this posture likely impairs their ability to take flight (as in Carr and Lima, 2012), its potential predation cost may be lower than the head-tuck posture because it does not interfere with visual and auditory capabilities. In addition, the leg-tuck posture likely conserves energy because birds expend less energy while sitting compared to standing (Tickle et al., 2012).

In the wild, peafowl prefer roosting at night in tall trees with thick understories. By sleeping at these sites, peafowl likely reduce their risk of predation from nocturnal predators (such as leopards and jungle cats; Trivedi and Johnsingh, 1996) that are active in both cold and warm temperatures (Majumder et al., 2011; Chattha et al., 2015). Future studies that examine whether peafowl adjust their thermoregulatory behavior relative to their exact sleeping site would be interesting. For example, peafowl may be more likely to exhibit the head-tuck posture if they are sleeping in relatively safe sites. Predator activity could also mediate their use of thermoregulatory postures. If predators are more active on colder nights (Bothma et al., 1994), for example, peafowl that exhibit the head-tuck posture on colder nights may be at higher risk than if they exhibited the head-tuck posture on warmer nights.

## MATERIALS AND METHODS

### Animal subjects

We examined the effect of two postures on thermoregulatory and antipredator behavior in adult Indian peafowl (*Pavo cristatus*) from a captive population (*n*=40) in West Lafayette, Indiana (40.450327°N, −87.052574°E) in the winter of 2016. The birds were originally captured from feral populations between 2008 and 2012. The head-tuck posture starts when the birds tuck their head under their wing and ends when the birds remove their heads from their wing; the leg-tuck posture starts when the birds tuck their legs and feet under their bodies by sitting and ends when they stand up (Fig. 1). The peafowl were individually marked with plastic and metal leg bands and housed in an outdoor enclosure (24.4 m×18.3 m×1.8 m). They were given food (corn and maintainer pellets) and water *ad libitum*. The study was approved by Purdue University's Animal Care and Use Committee (#1504001232).

### Bird temperature

#### Temperature sensors

We continuously monitored the temperature immediately surrounding the birds to assess the thermoregulatory role of the head-tuck and leg-tuck postures between January and March 2016. For each trial, we attached a temperature sensor (AXY-Depth, TechnoSmart, Rome, Italy that uses an ST Microelectronics LIS3DH sensor; 12×31×11 mm; 5.5 g; sample resolution: 19.6 ms^−2^; sample rate: 50 Hz) to either the head (11 females) or leg (the outside of the middle of their right tarsus; 8 females and 5 males) of the bird. The sensor was attached to the top of their head using velcro and glue (see Yorzinski et al., 2015 for a more detailed description of how the sensor was attached to the head) or attached to their leg using cable ties. The bird was transported to a testing cage (9 m×4.5 m) that was within the main enclosure by 18:30 Eastern Time (UTC-05:00). The testing cage was surrounded by black plastic so that the trial bird could not see the other birds in the main enclosure but could still hear them; no other birds were inside this testing cage during a trial aside from the trial bird. Prior to the start of this experiment, however, the door to this testing cage was open and birds were able to freely move between this area and the rest of their enclosure. The testing cage had a wooden roost (0.85 m high and 0.40 wide) with a horizontal support board that was in the middle of the roost (0.47 m high). A second temperature sensor was attached to this horizontal support board with velcro (birds were never in direct contact with this support board). The bird remained in the testing cage overnight and the sensors were removed the next day before the bird was returned to the rest of the flock. A camcorder (Bolide IR Bullet Camera) connected to a DVR (Swann DVR4-2600) recorded the bird as it spent the night on the roost. The video recordings were analyzed to determine the times at which the birds performed the head-tuck posture between sunset and sunrise. One of the head-tuck trials was dropped from the analyses because the bird never tucked its head at night. We determined the times at which the birds performed the leg-tuck posture between when the birds initially sat down for the night and stood up in the morning (the birds remained in the leg-tuck posture for over 95% of the night in all trials). Birds that hold their heads upright (while in the leg-tuck posture or not) often close their eyes at night (and are therefore likely resting or sleeping; Yorzinski and Platt, 2012); birds in the head-tuck posture likely have their eyes closed (and are also likely resting or sleeping).

The sensors did not appear to significantly impact the behavior of the birds. We examined the behavior – head scratching (scratching at their heads with their feet) and leg pecking (pecking at their legs with their beak) – of the birds during the first ten minutes after sunset (if the birds were not on the roost at that time, we examined their behavior during the first ten minutes after they ascended the roost). During the leg-tuck trials, only three of the 13 birds pecked at their legs (they each spent less than 10% of their time leg pecking). During the head-tuck trials, only one of the 10 birds scratched at her head (she spent 0.2% of her time head scratching). Furthermore, the birds frequently performed the head-tuck and leg-tuck postures while wearing the sensors (see Results) and the sensors did not appear to alter the way in which they performed these postures (the postures looked qualitatively similar regardless of whether they were wearing the sensors or not). Our previous work also showed that sensors attached to their heads did not impact head movement rates (Yorzinski et al., 2015).

#### Thermography

We measured the superficial temperatures of birds (3 males and 4 females) exhibiting the leg-tuck posture during the night between 20:00 and 00:30 Eastern Time (UTC-05:00) in February 2016. One researcher (J.L.Y.) identified a focal bird in the leg-tuck posture. The researcher touched the bird so that it would stand-up and then immediately took a thermal image (FLIR T420) of the bird. After five minutes, the researcher took another thermal image of the bird while it was still standing. The identity of the bird was determined immediately after the bird stood up and its leg bands were visible. The temperature of the beak, head, neck, body, wings, legs, and feet were determined from the thermal images; we used the spotmeter tool (FLIR Tools Software; version 5.4) to select these regions-of-interest and the software output their temperatures.

### Antipredator behavior

In order to determine whether the head-tuck posture limits antipredator behavior, we conducted approach trials during daylight between 10:30 and 18:30 Eastern Time (UTC-05:00) in February and March 2016. One researcher (J.L.Y.) identified a focal bird that was sitting with its head tucked (5 females and 4 males) or head upright (7 females and 2 males). Starting at least 9 m from the bird, the researcher slowly walked (approximately 0.33 mps) toward the front of the bird and stopped once the bird exhibited antipredator behavior. For birds in the head-tuck posture, antipredator behavior was scored when the birds untucked their heads; for birds with their heads upright, antipredator behavior was scored when the birds stood up. These are likely conservative estimates of antipredator behavior because birds with their heads tucked likely detected the threat before actually untucking their heads, and birds with their heads upright likely detected the threat before standing. The identity of the birds was determined at the end of each trial by approaching close enough that the birds stood up and their leg bands were visible. We measured the approach distance: the distance between the researcher and the bird at the time when the bird first exhibited antipredator behavior. Similar to other avian species (e.g. Geist et al., 2005), peafowl respond with avoidance behavior when humans approach (see Results) and therefore likely perceive humans as a threat.

### Measurements and statistical analysis

We assessed whether the percentage of time that birds spent in the head-tuck posture (total time that the head was tucked divided by the total time of the night) as well as the rate of the head-tuck posture (number of times that the head was tucked divided by the total time of the night; between sunset and sunrise) was related to the minimum ambient temperature (from the temperature sensor on the roost) and minimum wind speed (from a nearby weather station; http://iclimate.org; ACRE, West Lafayette) of the night; we ran these comparisons a second time examining the leg-tuck posture. Because the weather station only records weather data once every 30 min, we used our temperature sensors to obtain the ambient temperature data because the sensors recorded temperature continuously (sample rate: 50 Hz). We used custom Matlab scripts to extract the mean temperatures recorded by the temperature sensors during times when the birds exhibited the postures at night (444 bouts of birds exhibiting the head-tuck posture and 50 bouts of birds exhibiting the leg-tuck posture). We performed paired *t*-tests to examine whether the temperature of the birds' heads or legs (recorded from the temperature sensor on the bird) was different from ambient temperature (recorded from the temperature sensor on the roost) when the birds exhibited the head-tuck or leg-tuck posture, respectively. We also performed a mixed linear model (Proc MIXED in SAS; version 9.3; Cary, NC, USA) with bird identity as a random effect to assess whether the temperature difference between the birds' heads and ambient temperature (dependent variable) was related to the amount of time that the birds tucked their heads and ambient temperature (independent variables; only females were used in this study). Similarly, we performed another mixed linear model with bird identity as a random effect to assess whether the temperature difference between the birds' legs and ambient temperature (dependent variable) was related to the amount of time that the birds tucked their legs, ambient temperature, and sex of bird (independent variables). We included sex of the bird in the analysis because males are larger than females and this size difference could impact thermoregulation.

Based on the data from the thermal images, we performed a mixed linear model (Proc MIXED in SAS) to assess whether the temperature of the legs, feet or wings (control region) was influenced by timing (immediately after the bird stood versus 5 min after the bird stood), sex of the bird, wind speed, ambient temperature, and the interaction between wind speed and ambient temperature. Ambient temperature and wind speed were obtained from a nearby weather station (http://iclimate.org; ACRE, West Lafayette). We performed another mixed linear model to determine whether the temperature of the legs from this infrared thermography method (temperature immediately after the bird stood up from the leg-tuck posture) was similar to that using the temperature sensor (temperature while the bird was in the leg-tuck posture).

Also based on the data from the thermal images, we calculated heat loss from different morphological regions: beak, head, neck, body (including chest, abdomen, back and wings), legs, and feet. We calculated radiative heat exchange (Q_r_) and convective heat exchange (Q_c_) for each of the morphological regions using the method described by Tattersall et al. (2009):
(1)
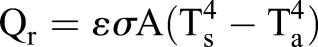

(2)
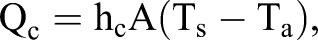


where T_s_ is the surface area of the morphological region (°K), T_a_ is the ambient temperature (°K), A is the surface area of the morphological region, ε is the combined emissivity of the object and the environment (assumed to be 0.97), σ is the Stephan-Boltzman constant (5.6703×10^−8^ W m^−2^ K^−4^), and h_c_ is the convective heat transfer coefficient for that morphological region (W m^−2^ °K^−1^). We then determined the whole body heat loss by summing the radiative heat exchange and convective heat exchange for all regions and calculated the percentage of heat loss for each region. We performed a mixed linear model to compare whether heat loss varied depending on timing [when the bird initially stood up (0 min) versus after standing (5 min)], morphological region (beak, head, neck, body, legs, or feet), sex of the bird, wind speed and the interaction between timing and morphological region; we performed contrasts to investigate which morphological regions varied in heat loss relative to timing.

Based on the data from the antipredator behavior experiment, we performed a Mann–Whitney test to examine whether the approach distance was related to whether the bird was sitting with its head tucked or head upright before the researcher approached. The ambient temperature was similar in upright (mean: −3.2°C; range: −7.7-16.5°C) and tucked (mean: −3.0°C; range: −7.9–17.4°C) trials and wind speed was also similar in upright (mean: −7.0 mps; range: 5–9.9 mps) and tucked (mean: −7.0 mps; range: 4.9–9.9 mps) trials. All statistical analyses were performed in SAS and means±s.e.m. are reported.
